# Secondary fungal infections in severe acute viral diseases: clinical features and underlying immune mechanisms

**DOI:** 10.3389/fmicb.2026.1780547

**Published:** 2026-03-06

**Authors:** Hanxin Li, Tong Wang, Tiandan Xiang, Ling Xu, Zhong Zheng, Xin Zheng

**Affiliations:** 1 Department of Infectious Diseases, Union Hospital, Tongji Medical College, Huazhong University of Science and Technology, Wuhan, China; 2 Joint International Laboratory of Infection and Immunity, Huazhong University of Science and Technology, Wuhan, China; 3 The First Affiliated Hospital of Soochow University, Suzhou, China

**Keywords:** acute viral diseases, host susceptibility, immune dysregulation, secondary fungal infections, virus-associated fungal infection

## Abstract

Secondary fungal infections are increasingly recognized as critical factors in the prognosis of severe acute viral infections, including influenza, SARS-CoV-2, Severe Fever with Thrombocytopenia Syndrome virus, and Dengue. This review outlines the clinical features of fungal complications, proposing a “virus-driven immune reprogramming” framework. It highlights how viral infections disrupt immune barriers, impair the Th17-IL-17 antifungal axis, attenuate platelet immune function, and involve unique pathogen interactions, creating a host immune microenvironment that is more susceptible to fungal invasion. Understanding these immune-injury mechanisms underscores the clinical importance of earlier surveillance of secondary fungal disease and informs the development of mechanism-guided therapeutic approaches to improve patient outcomes.

## 1 Introduction

Severe acute viral diseases constitute a major global health burden, not only because of direct viral pathogenicity but also due to their strong propensity to predispose affected individuals to secondary infections. Viral pathogens such as influenza viruses (MacIntyre et al., 2018), severe acute respiratory syndrome coronavirus 2 (SARS-CoV-2) (Fehér et al., 2022), severe fever with thrombocytopenia syndrome virus (SFTSV) (Ge et al., 2022), and dengue virus (Premaratna et al., 2015; Lin et al., 2023) have been increasingly associated with secondary infectious complications involving both bacterial and fungal pathogens. Clinical and epidemiological studies consistently demonstrate that secondary infections substantially aggravate disease severity, accelerate clinical deterioration, prolong hospitalization, and markedly increase mortality in patients with severe acute viral diseases (Alshrefy et al., 2022). Notably, these superinfections are no longer confined to individuals with classical immunosuppressive conditions, suggesting that virus-induced immune dysregulation itself plays a central role in shaping host susceptibility to opportunistic and invasive pathogens.

Among secondary infections in severe acute viral diseases, fungal co-infections present unique clinical challenges due to their rapid progression, diagnostic difficulty, and high mortality (Suzuki et al., 2007; Zhang et al., 2022; Lin et al., 2023; Feys et al., 2024). Reported incidences of secondary fungal infections vary widely across clinical settings, ranging from <5% in general hospitalized cohorts to over 20%–30% among critically ill or intensive care units (ICUs) patients, depending on the viral pathogen, host factors, and diagnostic criteria (Lu et al., 2024; Zuniga-Moya et al., 2024; Hu et al., 2025; Xia et al., 2025). Respiratory viruses are particularly prone to being complicated by secondary fungal infections, with *Aspergillus* species being the most commonly reported pathogens. In contrast, secondary fungal infections in SFTSV and dengue virus infections appear less frequent but clinically consequential. Available cohort studies and case series indicate that fungal co-infections in SFTSV predominantly occur in patients with severe disease and are associated with substantially increased mortality (Wang et al., 2024), although precise incidence estimates remain limited. Dengue-associated secondary fungal infections are rare at the population level but have been increasingly reported as rapidly progressive and often fatal complications, even in immunocompetent hosts (D J et al., 2024).

Against this background, the World Health Organization (WHO) now classifies azole-resistant *Aspergillus fumigatus* and *Candida glabrata* as high-priority fungal pathogens, reflecting elevated risks of treatment failure and mortality (WHO fungal priority pathogens list to guide research, development and public health action, World Health Organization [WHO], 2025). Consequently, early recognition and timely intervention of secondary fungal infections represent a critical yet underexplored clinical challenge. Most existing studies on fungal diseases have focused on patients with chronic immunosuppression, whereas the risk factors and immune mechanisms driving fungal superinfections in severe acute viral diseases remain poorly defined (Castón-Osorio et al., 2008). Although certain immune pathways may overlap with those observed in chronic viral infections such as HIV and cytomegalovirus (CMV), acute viral infections induce a distinct and dynamic immune landscape characterized by simultaneous hyperinflammation and compensatory immunosuppression. In this review, we critically examine the emerging evidence on virus-associated secondary fungal infections and propose an integrated conceptual framework that links virus-induced immune reprogramming to fungal susceptibility across different acute viral diseases, with the goal of informing earlier risk stratification and improving clinical management.

## 2 Clinical features of secondary fungal infections following acute viral infections

### 2.1 Respiratory viruses with secondary fungal infection

Respiratory viral infections currently represent the most extensively investigated clinical setting for understanding acute virus–fungus interactions.

Influenza A virus (IAV), a negative-sense single-stranded RNA virus of the Orthomyxoviridae family, causes a highly contagious respiratory illness that may rapidly progress to severe pneumonia and fatal complications, particularly among high-risk or critically ill individuals (Oliveira et al., 2017). Invasive aspergillosis was first identified as a major secondary complication during the 2009 H1N1 influenza pandemic (Lat et al., 2010). Subsequent studies have demonstrated that *Aspergillus* co-infection is independently associated with excess mortality in severe influenza (Duan et al., 2020; Coste et al., 2021). Cumulative evidence further suggests that approximately 10% of critically ill influenza patients develop influenza-associated pulmonary aspergillosis (IAPA), with pooled mortality rates reaching 50%–54%, indicating its significant clinical impact (Shi et al., 2022; Lu et al., 2024). Besides *Aspergillus*, other opportunistic molds may also complicate influenza infection. Influenza-associated mucormycosis (IAM) typically presents as pulmonary disease, with systemic corticosteroid therapy being the major predisposing factor (Ahmadikia et al., 2021). Influenza-associated cryptococcosis is exceedingly rare; only two cases of cryptococcal meningitis were reported during the 2009 H1N1 pandemic, with an additional case of disseminated disease following A/H7N9 infection (Hosseinnezhad and Rapose, 2012; Gupta et al., 2015; Huang et al., 2019).

This clinical pattern of respiratory virus-associated secondary fungal infection became even more prominent during the COVID-19 pandemic, contributing substantially to mortality among critically ill patients. COVID-19-associated pulmonary aspergillosis (CAPA) occurs in approximately 10.2% of patients in intensive care units (ICUs), with some cohorts reporting incidences as high as 19% (Mitaka et al., 2021; Bergmann et al., 2023). These infections typically arise within a specific window–3–10 days after symptom onset–and mortality frequently exceeds 50% (Schauwvlieghe et al., 2018; Xia et al., 2025). Among older adults, nearly one-quarter develop secondary infections, with CAPA being the most common; mortality may exceed 40% when acute respiratory distress syndrome (ARDS) is present (Nasir et al., 2020; Chong and Neu, 2021; Chong et al., 2022).

Invasive candidiasis represents the second most common fungal complication in COVID-19, with candidemia predominating in ICUs (Basile et al., 2022). Moreover, hospitalized patients-particularly those of advanced age, with multiple comorbidities, or underlying immunosuppression-are predisposed to oropharyngeal candidiasis due to the combined effects of mucosal barrier disruption and virus-induced immune dysregulation (Salehi et al., 2020; Zhang et al., 2020). Despite geographic variation, COVID-19-associated mucormycosis (CAM) exhibits consistently high mortality, often exceeding 50%, especially in patients with metabolic disorders (Pal et al., 2021; Balushi et al., 2022). Unlike IAM, which primarily involves the lungs, CAM most commonly manifests as rhino-orbito-cerebral disease, with pulmonary and gastrointestinal involvement being less frequent (Ahmadikia et al., 2021; Chakravarty et al., 2022). COVID-19-associated cryptococcosis is sporadic, most often presenting as disseminated cryptococcemia, followed by pulmonary infection or meningitis (Passarelli et al., 2020; Cafardi et al., 2021; Ghanem and Sivasubramanian, 2021).

*Pneumocystis jirovecii* pneumonia (PCP), historically associated with advanced HIV infection, is also observed in COVID-19–predominantly among individuals with pre-existing immunosuppression (Chong et al., 2021), though cases have also been reported in patients without known immune defects, which are often with atypical clinical features that delay diagnosis and are associated with poor outcomes (Yu and Yang, 2024; Xu et al., 2025). These observations suggest that SARS-CoV-2 infection may unmask or exacerbate susceptibility to fungal pathogens.

Collectively, influenza viruses and SARS-CoV-2 constitute the most typical respiratory viral backgrounds for secondary fungal infections. These viruses generate a transient yet highly permissive window of susceptibility that significantly increases host vulnerability to *Aspergillus*, Mucorales, *Candida*, *Pneumocystis*, and other fungal pathogens. This pattern not only manifests as high-incidence CAPA and ICU-associated candidemia, but also as rare yet highly lethal conditions such as mucormycosis and cryptococcosis. Overall, the temporal characteristics, concentration among high-risk populations, and severe clinical outcomes of respiratory virus-associated fungal complications underscore the need for proactive, risk-stratified surveillance early in viral illness, rather than reactive diagnosis following clinical deterioration. This disease model also provides important clinical support for understanding the immune mechanisms through which acute viral infections confer fungal susceptibility.

### 2.2 Arthropod-borne viruses with secondary fungal infection

In addition to respiratory viruses such as influenza that exert pathogenicity primarily within the airway, arthropod-borne viral diseases–including SFTS and dengue–can also lead to secondary fungal infections and adverse clinical outcomes.

Severe fever with thrombocytopenia syndrome virus is a tick-borne acute viral disease endemic to East Asia, characterized by high fever, thrombocytopenia, leukopenia, gastrointestinal symptoms, hepatic dysfunction, coagulopathy, and progressive multi-organ involvement. Reported case fatality rates range from 5% to 30%, depending on disease severity and geographic region (Kobayashi et al., 2020). In recent years, SFTS has increasingly been recognized as a viral syndrome with a pronounced propensity for secondary infectious complications, particularly invasive fungal infections (IFIs), which further accelerate clinical deterioration and significantly worsen prognosis.

The reported incidence of secondary IFIs in patients with SFTS ranges from 12% to 32%, with most cases emerging during the second to third week after disease onset, coinciding with profound immune dysregulation and sustained cytopenias (Song L. et al., 2023; Zuo et al., 2023; Yao et al., 2024). *Candida* and *Aspergillus* species represent the predominant fungal pathogens (Ge et al., 2022; Song H. et al., 2023), with *Aspergillus fumigatus* being the most frequently isolated organism, commonly detected in respiratory specimens and strongly associated with invasive pulmonary manifestations (Hu et al., 2025). Among SFTS patients admitted to ICUs, up to 56% develop invasive pulmonary aspergillosis, with a median onset approximately 8 days after ICU admission, and fungal co-infection is associated with worse clinical outcomes (Bae et al., 2020). Although *Candida* isolation has been reported in some cases, current evidence does not consistently support bloodstream infection as the predominant presentation. Rare reports of *Aspergillus fumigatus* detected in cerebrospinal fluid further suggest the possibility of multi-organ dissemination (Li et al., 2025).

Dengue is a mosquito-borne acute viral disease endemic in tropical and subtropical regions and is clinically characterized by fever, rash, bleeding tendency, thrombocytopenia, plasma leakage, and–in severe forms–shock and multi-organ dysfunction (Botez and Doughty, 2014). Although dengue is not classically categorized as an immunosuppressive disease, accumulating reports document severe secondary fungal infections across a range of clinical presentations. Post-dengue mucormycosis, for example, has been repeatedly described–typically arising during the convalescent or immune-rebound phase–and often manifests as rhino-orbital or sino-nasal disease even in individuals previously considered immunocompetent (Singhal et al., 2022; D J et al., 2024). Mechanistic studies suggest that profound thrombocytopenia, capillary leakage, metabolic acidosis, and tissue hypoxia in severe dengue may collectively create a microenvironment conducive to Mucorales invasion and expansion (Popli et al., 2024). Invasive pulmonary aspergillosis has also been documented in dengue hemorrhagic fever and among critically ill patients requiring mechanical ventilation (Lin et al., 2023; Masud et al., 2025), while disseminated candidiasis has been observed in dengue shock syndrome–likely associated with increased intestinal permeability and microbial translocation (Suzuki et al., 2007).

Compared with respiratory virus–associated fungal disease, SFTS and dengue exhibit several distinct features: both viruses can rapidly induce systemic inflammatory responses, coagulation dysfunction, and tissue hypoperfusion, leading to sepsis, thereby markedly enhancing host susceptibility to invasive fungal pathogens such as Mucorales, *Aspergillus*, and *Candida*. Moreover, the temporal window for fungal infection generally appears later than in respiratory viral infections and is more frequently characterized by extra-pulmonary and multi-organ dissemination.

Collectively, these clinical observations highlight a unifying feature across acute viral diseases: a transient but profound susceptibility to invasive fungal infection, even in the absence of classical immunosuppression. To understand this vulnerability, it is essential to examine how viral infections reshape host immunity. The next section therefore, focuses on immune mechanisms underlying virus-associated fungal susceptibility.

## 3 Immune mechanisms of secondary fungal infections following acute viral infections

### 3.1 Host-related risk factors

The high incidence of secondary fungal infections in the context of severe acute viral diseases is closely linked not only to virus-induced immune dysregulation but also to host baseline characteristics and clinical management during hospitalization. Under conditions of acute critical illness, patients frequently require intensive supportive care, including invasive procedures, prolonged ICU stays, mechanical ventilation, broad-spectrum antibiotic exposure, and treatment with systemic corticosteroids or other immunomodulatory agents. These factors collectively compromise host defenses and markedly increase susceptibility to fungal invasion. Table 1 summarizes major host-related risk factors associated with secondary fungal infections across four representative acute viral diseases–namely, influenza, COVID-19, SFTS, and dengue. Notably, many of these risk factors reflect transient immune dysfunction and treatment-related immunomodulation rather than classical chronic immunosuppression, highlighting a distinct clinical context in which invasive fungal diseases may emerge.

**TABLE 1:** Fungal co-infections, underlying diseases, and risk factors in humans.

Underlying disease/infection	Fungal infection	Risk factors associated with co-infection	References
Influenza	*Aspergillus*	Liver cirrhosis; influenza A(H1N1)pdm09 subtype; hematologic malignancy; vasopressor requirement.	Coste et al., 2021
		Asthma and days of mechanical ventilation.	Waldeck et al., 2023
		Risk prediction model: Asper-PreSS model – accumulated steroid dose > 200 mg; CD4^+^ T-cell < 200/μl; dry rales; refractory fever ≥ 3 days, et al.	Huang et al., 2020
	Mucormycosis	Neutropenia; immunosuppression; uncontrolled diabetes; prolonged steroid therapy.	Ahmadikia et al., 2021
COVID-19	*Aspergillus*	Invasive respiratory support; tocilizumab therapy; age.	Prattes et al., 2022
		Chronic liver disease; hematological malignancies; COPD; cerebrovascular disease; invasive mechanical ventilation; renal replacement therapy; IL-6 inhibitors; corticosteroids.	Gioia et al., 2024
	Mucormycosis	Diabetes mellitus; high-dose corticosteroid therapy.	Ramaswami et al., 2021; Sen et al., 2021; Tavakolpour et al., 2022
		Smoking.	Tavakolpour et al., 2022
	Candidiasis	Corticosteroid therapy; broad-spectrum antibiotics therapy; nasal corticosteroid spray.	Silva et al., 2021
		Mechanical ventilation.	Babamahmoodi et al., 2022
	Cryptococcosis *Pneumocystosis*	Systemic corticosteroid.	Chan et al., 2022
		Tocilizumab; baricitinib.	Chastain et al., 2022
		Severe lymphocytopenia (<1000 cells/mm^3^); CD4^+^ T-cell < 200 cells/mm; long-term immunosuppressants; invasive mechanical ventilation.	Cattaneo et al., 2023
SFTS	*Aspergillus*	CD4^+^ T-cell < 68 cells/mm^3^ and CD8^+^ T-cell < 111 cells/mm^3^; IL-6 > 99 pg/ml and IL-10 > 111 pg/ml; BNP > 500 pg/ml.	Hu et al., 2021
		Neurological complications; high SFTSV RNA loads; low WBC, PLT, ALB, GLB; elevated cTNI.	Wang et al., 2024
		Advanced age; petechia; hemoptysis; tremor; low albumin levels; prolonged APTT; ICU admission; glucocorticoid use; IVIG; prolonged hospitalization.	Yao et al., 2024
	Unclassified fungal infections (*Aspergillus*, candidiasis, yeast infections, dermatophytosis)	CRRT; ICU transfer; antifungal use; elevated LDH.	Hu et al., 2025
Dengue	Disseminated candidiasis Trichosporon	Intestinal translocation.	Suzuki et al., 2007
		Indwelling catheter.	Urs et al., 2018; Sachan et al., 2022
	Rhino-orbital mucor mycosis	Chronic sinusitis; deviated nasal septum.	D J et al., 2024.

COPD, chronic obstructive pulmonary disease; IL-6, interleukin-6; WBC, white blood cell; PLT, blood platelet; ALB, albumin; GLB, globulin; cTNI, cardiac troponin I; BNP, brain natriuretic peptide; APTT, activated partial thromboplastin time; IVIG, intravenous immunoglobulin; CRRT, continuous renal replacement therapy; LDH, lactate dehydrogenase.

### 3.2 Disruption of host immune barriers

The mucosal barrier is the first line of defense for the host, effectively preventing pathogen invasion and maintaining homeostasis between the internal and external environments. In the immune system, the integrity of the mucosal barrier is crucial for defending against fungal infections (Zhang et al., 2025). However, viral infections can disrupt this barrier through various mechanisms, increasing susceptibility to fungi. Respiratory viruses, exemplified by influenza and SARS-CoV-2, enter epithelial cells via sialic acid receptors and ACE2 receptors, respectively. The viral replication process within these cells leads to necrosis and apoptosis of epithelial cells, directly compromising the integrity of the physical barrier (Hui et al., 2022). Additionally, the envelope (E) protein of SARS-CoV-2 downregulates tight junction proteins such as ZO-1, occludin, and claudins, causing widened intercellular gaps and increased epithelial permeability (Shepley-McTaggart et al., 2021; Xu et al., 2024). Viral infections can also suppress goblet cell secretion of mucin (MUC5AC) through an IL-13-dependent pathway, further weakening the protective function of the mucus layer (Morrison et al., 2022). Finally, the inflammatory cascade triggered by pathogen invasion, including neutrophil recruitment and the production of reactive oxygen species (ROS), exacerbates tissue damage (Tay et al., 2020), leading to epithelial denudation and exposure of basement membrane components such as collagen, laminin, and fibronectin, which serve as binding substrates for fungal adhesion (e.g., FbpA of *Aspergillus*, Als3 of *Candida*) (Feys et al., 2022). These alterations collectively form the pathological axis of “virus-induced damage–fungal colonization.”

In patients with dengue virus infection, the dengue virus non-structural protein 1 (NS1) has been shown to cross-react with human endothelial cells, triggering NF-κB–regulated immune activation and nitric oxide (NO)–mediated apoptosis, thereby inducing endothelial cell injury and apoptosis (Lin et al., 2002; Chen et al., 2013). Beyond systemic endothelial damage, multiple studies have demonstrated significant intestinal mucosal injury in patients with dengue infection (Vejchapipat et al., 2006). Case reports suggest that secondary candidemia associated with dengue is typically caused by common gut-derived *Candida* species (Suzuki et al., 2007). Moreover, previous investigations of secondary bacterial infections following dengue have revealed that the causative pathogens predominantly originate from the gut, with members of the Enterobacteriaceae family, *Enterococcus* spp., and *Streptococcus* spp. being more prone to translocation than other Gram-positive or anaerobic bacteria (Steffen et al., 1988). Collectively, these findings support an emerging concept: a key mechanism underlying dengue-associated disseminated fungal infection may be intestinal mucosal barrier disruption leading to microbial translocation.

### 3.3 Immune cell injury and dysfunction

#### 3.3.1 CD4^+^ T-cell depletion and Th17 impairment

In immunocompetent individuals, invasive fungal infections (IFIs) are rare, primarily due to the strong antifungal defenses provided by the adaptive immune system and the protection offered by being warm-blooded (Casadevall, 2022). CD4^+^ T-cells represent a central component of adaptive antifungal defense; impairment of their differentiation toward the Th17 lineage compromises IL-17-mediated CXCL chemokine production and neutrophil recruitment–both essential for effective antifungal immunity (Korn et al., 2009). In classical immunosuppressive viral infections such as HIV and cytomegalovirus, the risk and pathogen spectrum of invasive fungal disease (IFD) strongly correlate with the extent of CD4^+^ T-cell depletion and exhibit pathogen patterns aligned with distinct CD4^+^ thresholds (Park et al., 2009; Patel et al., 2012; Jarvis et al., 2013; Skipper et al., 2020).

Clinical observations further demonstrate that reduced CD4^+^ T-cell counts are strongly associated with secondary following a range of acute viral infections. In SFTS-associated pulmonary aspergillosis (SAPA), influenza-associated pulmonary aspergillosis (IAPA), and COVID-19-associated *Pneumocystis jirovecii* pneumonia, decreased CD4^+^ T-cell levels correlate not only with disease severity but also with the incidence of secondary fungal disease (Huang et al., 2020; Hu et al., 2021; Cattaneo et al., 2023; Zuo et al., 2023). Mechanistically, inflammation-driven activation-induced apoptosis is considered a major contributor to T-cell loss in severe COVID-19 and influenza, but emerging virological evidence suggests that SARS-CoV-2 may additionally interact with CD4 molecules and infect CD4^+^ T helper cells, potentially amplifying T-cell depletion and functional exhaustion (Nichols et al., 2001; Diao et al., 2020; Brunetti et al., 2023; Shouman et al., 2024). Furthermore, highly pathogenic influenza viruses (e.g., H5N1) may activate plasmacytoid dendritic cells to express Fas ligand, amplifying systemic inflammation and leading to significant loss of CD4^+^ and CD8^+^ lymphocytes (Boonnak et al., 2014). Beyond inflammation-driven apoptosis, additional virus-specific mechanisms further disrupt T-cell homeostasis. SFTSV directly infects the thymus, impairing T-cell development and output (Ma et al., 2025), whereas dengue virus does not consistently induce quantitative CD4^+^ T-cell depletion but alters cytokine signaling networks and T-cell homeostatic cues, thereby affecting CD4^+^ T-cell functionality, trafficking, and tissue distribution (Singla et al., 2016; Khanam et al., 2022).

In severe COVID-19 and influenza, marked peripheral CD4^+^ and CD8^+^ T-cell reductions represent among the earliest and most consistent immune abnormalities, with the degree of lymphopenia closely mirroring clinical severity (Chen et al., 2020; Qian et al., 2020). Similarly, early-phase CD4^+^ T-cell depletion is detectable in patients with severe SFTS, supporting the hypothesis that apoptosis- and exhaustion-driven CD4^+^ T-cell loss is a key determinant of susceptibility to secondary fungal infection (Song et al., 2024). Collectively, these findings indicate that acute viral infections converge through multiple pathways to impair CD4^+^ T-cell–mediated antifungal immunity, thereby generating a transient yet highly permissive window for the development of secondary invasive fungal disease.

#### 3.3.2 Platelet dysfunction and loss of antifungal immunity

Platelets have traditionally been regarded as key effector cells of the coagulation system; however, increasing evidence indicates that they also exert critical immunoregulatory and antimicrobial functions, positioning them as multifunctional cells at the intersection of immunity and hemostasis (Nicolai et al., 2024). In antifungal defense, platelets play active and multifaceted roles: they can directly damage *Candida albicans* through degranulation and antimicrobial peptide release (Schultz et al., 2020), enhance neutrophil recruitment, and suppress *Aspergillus* germination and hyphal growth (Speth et al., 2013; Launder et al., 2024). In thrombocytopenia-associated viral infections, including SFTS and dengue, impaired platelet-mediated antifungal responses may represent a plausible yet incompletely defined mechanism underlying the increased propensity for invasive fungal infections in these patients.

### 3.4 Inflammatory homeostasis imbalance and immune dysregulation

#### 3.4.1 Dysregulated interferon signaling

The interferon signaling cascade is not only a central axis of antiviral immunity but also a critical upstream regulatory hub maintaining mucosal and systemic antifungal immune homeostasis. While type I and type II interferons are crucial for inhibiting viral replication and activating antiviral immune responses, excessive or prolonged activation can disrupt immune networks and impair the host’s ability to clear fungal infections. Type I interferons suppress inflammasome activity and induce interleukin-10 (IL-10), thereby reducing the availability of pro-IL-1α and pro-IL-1β (Guarda et al., 2011). Given the central role of IL-1 signaling in driving Th17 differentiation, this suppression weakens IL-17-dependent neutrophil recruitment and mucosal antifungal defenses (Griffiths et al., 2021). As a result, despite preserved circulating neutrophil counts, their phagocytic capacity, ROS production, and neutrophil extracellular trap (NET) formation are functionally impaired (Urban and Backman, 2020).

Dysregulated type II interferon responses similarly contribute to antifungal susceptibility. In IAPA, an early surge of interferon-γ (IFN-γ) suppresses Th17 responses and impairs macrophage-mediated fungal clearance. In addition, IFN-γ disrupts macrophage-neutrophil coordination, interferes with phagolysosomal maturation, and reduces ROS-dependent killing, all of which contribute to *Aspergillus* invasion (Seldeslachts et al., 2024). Recent evidence further implicates disruption of a B1a–natural IgG neutrophil axis as an additional mechanism linking interferon dysregulation to functional neutrophil insufficiency in viral- and steroid-associated aspergillosis (Sarden et al., 2022).

#### 3.4.2 IL-10–dominated immune regulation

Interleukin-10 is an important anti-inflammatory cytokine that limits host tissue damage by suppressing excessive pro-inflammatory responses and modulating both innate and adaptive immunity (Rojas et al., 2017). However, dysregulated or prolonged IL-10 production can disrupt immune balance, increasing susceptibility to secondary fungal infections. Experimental studies highlight the dual role of IL-10 in fungal immunity: IL-10-deficient mice exhibit increased susceptibility to *Aspergillus* infection (Clemons et al., 2000), whereas IL-10 knock-out improves survival and viral clearance during influenza virus infection (Sun et al., 2010). This suggests that the upregulation of IL-10 during antiviral responses helps control inflammation, but it also weakens antifungal immunity, making the host more susceptible to fungal infections. Clinical studies have also reported elevated IL-10 levels in patients with CMV-associated chronic pulmonary aspergillosis and in those with secondary *Aspergillus* infections following SFTS, often accompanied by increased IL-6 levels (Hu et al., 2021; Huang et al., 2022).

According to classical immunology, IL-10 acts as a compensatory regulatory mechanism in response to excessive inflammation, primarily produced by macrophages, monocytes, dendritic cells, and activated T cells. The high inflammatory state induced by severe viral infections can drive IL-10 transcription through the STAT3-dependent pathway to reduce tissue damage. Additionally, virus-specific effector T cells can acquire the ability to produce IL-10 during their initial activation in highly inflammatory environments. In the case of influenza, IL-10 induction is regulated by IL-12 and IL-27-mediated T-cell priming signals, which help alleviate lung immune pathology (Sun et al., 2009). In dengue, viral proteins like NS1 directly stimulate IL-10 production by monocytes and macrophages (Adikari et al., 2016), whereas in SFTS, the NSs protein activates the NF-κB pathway via TPL2, increasing IL-10 expression and contributing to immune modulation (Choi et al., 2019).

Furthermore, some viruses actively exploit this immune regulatory mechanism by encoding viral IL-10 homologs (vIL-10) that mimic host IL-10 signaling (Ouyang et al., 2014). For example, cytomegalovirus-derived CMVIL-10 competitively binds to the IL-10 receptor (IL-10R) (Jones et al., 2002) and downregulates MHC class I and II expression (Spencer et al., 2002), suppresses dendritic cell activation, and impairs antigen presentation (Chang et al., 2004, 2009; Avdic et al., 2014). Epstein-Barr virus-derived EBVIL-10 retains immunosuppressive functions but lacks immunostimulatory activity due to altered IL-10R affinity (Yoon et al., 2012). Notably, recent studies suggest that vIL-10 has an unexpectedly limited role in antifungal immunity, as it preferentially suppresses antiviral and inflammatory signals (Heilig et al., 2025). The selective regulation by vIL-10 may partly explain the inconsistency of IL-10’s predictive role in fungal infections observed in clinical studies.

**FIGURE 1:**
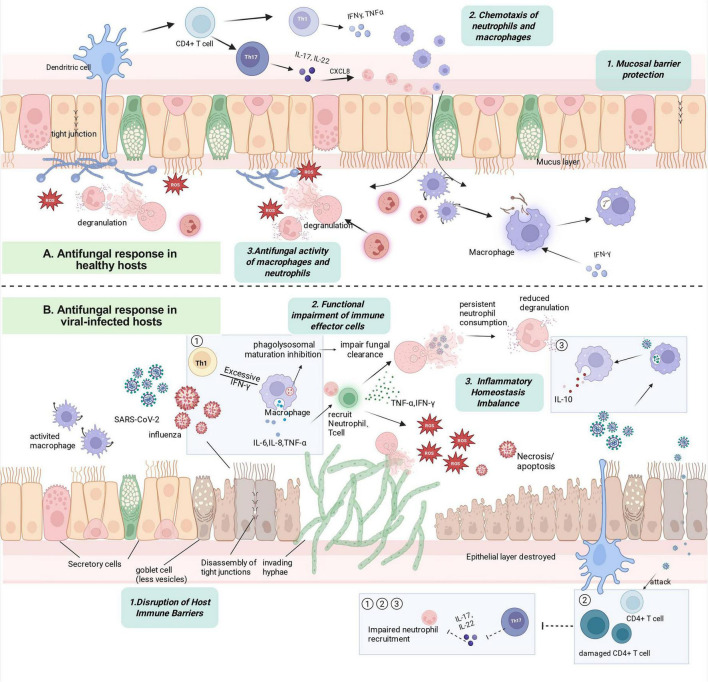
Overview of the mechanistic processes linking respiratory viral infection to secondary fungal infection, this schematic focuses on representative mechanisms observed with respiratory viruses (Influenza A, SARS-CoV-2). **(A)** Antifungal response in healthy hosts. **(B)** Antifungal response in viral-infected hosts. Key mechanistic processes include: (1) disruption of host immune barriers; (2) exhaustion and functional impairment of immune cells, including neutrophils, macrophages, and T cells, characterized by functional neutropenia and compromised macrophage antifungal effector function. (3) inflammatory homeostasis imbalance. Viral infections disrupt the dynamic equilibrium between pro-inflammatory and anti-inflammatory responses. Activated macrophages and neutrophils release excessive inflammatory cytokines, such as IL-6 and TNF-α, driving cytokine storm–mediated tissue injury. In parallel, overproduction of immunosuppressive mediators, such as IL-10, dampens antifungal effector functions. This coexistence of hyperinflammation and immunosuppression creates a permissive environment for secondary fungal infection. ① ② ③ represent three mechanistic dimensions of disruption to the Th17–IL-17–neutrophil antifungal immune axis: ① excessive IFN-γ weakens macrophage-mediated fungal clearance (Seldeslachts et al., 2024), ② SARS-CoV-2 and influenza viruses directly or indirectly reduce CD4^+^ T-cell levels, ③ High inflammatory state induced IL-10 production. Image created with Biorender.com.

The immune dysregulation caused by acute viral infections generally points to a central axis: the Th17-IL-17-neutrophil antifungal immune axis. On one hand, lymphopenia and CD4^+^ T cell depletion observed in COVID-19, SFTS, and severe dengue directly reduce the basis for Th17 differentiation. On the other hand, dysregulated interferon signaling inhibits IL-1-dependent Th17 differentiation and impairs neutrophil function. Meanwhile, IL-10-driven immune regulation weakens IL-17-mediated mucosal defense. The combined effects of these factors create a transient but highly susceptible window for secondary invasive fungal infections, which can occur even in the absence of classical immunosuppression. Figure 1 summarizes the mechanistic processes linking respiratory viral infection to secondary fungal infection.

### 3.5 Direct virus–fungus interactions and pathogen synergy

Beyond host immune dysregulation, direct molecular interactions between viruses and fungi can further exacerbate disease progression. In acute respiratory viral infections, IAV impairs lysosomal maturation in neutrophils and macrophages, thereby reducing intracellular killing of *Aspergillus conidia* (Liu et al., 2022). Consistent with these functional defects, transcriptomic analyses from patients with IAPA and CAPA reveal coordinated downregulation of gene programs involved in fungal recognition, phagolysosomal processing, and microbial clearance (Feys et al., 2022). In addition, purified IAV particles can bind sialic acid residues on the surface of *Aspergillus fumigatus*, directly stimulating hyphal growth and promoting fungal proliferation independent of host immunity (König et al., 2024).

Together, these findings underscore that virus-associated fungal infections arise from bidirectional, pathogen-specific interactions rather than linear consequences of host immunosuppression alone. Importantly, virus-driven immune dysregulation may precede and facilitate the progression of fungal exposure or colonization to invasive disease by weakening key antifungal effector functions, including phagocyte-mediated recognition and intracellular killing. Once fungal growth begins, direct pathogen synergy, such as virion-fungus binding that accelerates hyphal expansion, may further increase fungal burden and tissue injury, amplifying local inflammation and further compromising antifungal defense. In this framework, direct virus-fungus interactions act as an amplifier on top of leukocyte dysfunction and cytokine imbalance, reinforcing the proposed immune vulnerability window.

## 4 Conclusion

The occurrence of invasive fungal diseases (IFDs) has traditionally been attributed to primary immunodeficiency or acquired immunosuppression. Congenital antifungal immunity defects, such as CARD9 gene deficiency, clearly demonstrate how impaired innate immune signaling predisposes the host to severe fungal infections through defects in fungal recognition, impaired Th17 differentiation, and insufficient neutrophil recruitment or fungicidal activity (Drummond et al., 2018; Chiriaco et al., 2019). Acquired immunosuppression, including neutropenia, prolonged corticosteroid use, solid organ transplantation, and hematologic malignancies, is similarly closely associated with a high incidence of IFDs (Castón-Osorio et al., 2008; Wiedermann, 2021).

However, emerging evidence from acute viral infections challenges this static framework. Acute virus-associated secondary fungal infections should no longer be viewed as passive opportunistic infections, but rather as a result of virus-driven immune reprogramming. This reprogramming is characterized by the coexistence of excessive inflammatory activation and compensatory immunosuppression induced by acute viral infection, in which cytokines such as IL-6, TNF-α, and IL-10 play pivotal roles in shaping the antiviral response. These alterations ultimately impair key antifungal immune axes, particularly the CD4^+^ T cell–Th17–neutrophil immune axis. Additional contributing mechanisms include platelet dysfunction and pathogen-level interactions, which may further promote fungal susceptibility. Together, these processes give rise to a highly susceptible “immune vulnerability window.” This window may be further amplified by host-related factors such as nutritional status, metabolic dysregulation, iatrogenic immunosuppression [e.g., corticosteroid use (Ahmadikia et al., 2021)], or immune-independent pathogen interactions (e.g., direct binding between influenza A virus particles and *Aspergillus fumigatus*), Consequently, invasive fungal disease may develop even in patients with acute viral infections who would not typically meet the criteria for classical immunosuppression. A similar temporal pattern has been observed in sepsis, in which an initial hyperinflammatory response is frequently followed by compensatory immunosuppression characterized by lymphocyte apoptosis, reduced monocyte HLA-DR expression, and impaired antigen presentation, resulting in increased susceptibility to secondary infections (Hotchkiss et al., 2013, 2016; Delano and Ward, 2016). Recognizing this dynamic immune trajectory has important clinical implications. It suggests that clinical management strategies must shift from solely treating fungal infections to early identification of the immune phenotypes, dynamic monitoring of immune axis damage, and the development of more targeted intervention pathways based on immune mechanisms.

The mechanisms of virus–fungus interactions also suggest that future treatment strategies should move beyond the traditional approach of simply enhancing antifungal therapy and shift toward mechanism-guided precision interventions. Strategies targeting the blockade of fungal invasion pathways have shown protective effects in preclinical models. Therapeutic approaches targeting the CotH–GRP78 binding axis in Mucorales (Gebremariam et al., 2014, 2019), as well as recent promising antifungal activity demonstrated by monoclonal antibodies (e.g., VX-01) derived from anti-CotH IgG1 (Gu et al., 2025), mark a potential paradigm shift toward therapies that block fungal invasion rather than fungal viability alone.

At the same time, immunomodulatory treatments may offer adjunctive value in certain contexts. Adjunctive IFN-γ therapy has shown efficacy in some invasive fungal infections (IFIs), including transplant-associated fungal disease and HIV-related cryptococcal meningitis (Armstrong-James et al., 2010; Lehrnbecher et al., 2011; Jarvis et al., 2012). However, increasing evidence indicates that the therapeutic impact of immune modulation is highly dependent on the timing of viral infection and the immune status of the host (Seldeslachts et al., 2024), highlighting the potential risks of indiscriminate immune enhancement and the importance of timing in immune modulation.

Despite these promising advances, the translation of mechanism-guided antifungal and immunomodulatory strategies into routine practice remains challenging. Reliable biomarkers are needed to identify patients entering a high-risk immune phase. Longitudinal monitoring of lymphocyte subsets, CD4^+^ T-cell counts, and interferon signatures may help stratify patients according to their immune trajectory. Integrating immune profiling into routine clinical assessment could facilitate timely initiation of antifungal prophylaxis or immunomodulatory therapy in selected high-risk individuals. Moreover, most current evidence derives from respiratory viral infections, while data in non-respiratory viral diseases such as SFTS and dengue remain sparse. Large-scale prospective studies are needed to validate risk stratification models and to define the safety and efficacy of immune-targeted therapies in these settings. Until such data become available, precision antifungal strategies should be applied cautiously and tailored to the clinical and immunological context.

Overall, this review emphasizes that virus-associated invasive fungal infections arise from complex and dynamic immune perturbations, rather than simple immunosuppression. Important knowledge gaps remain, particularly regarding the temporal dynamics of virus-driven immune reprogramming, the role of direct virus-fungus interactions, and biomarker-guided identification of the immune vulnerability window. Addressing these challenges will require integrated mechanistic, translational, and prospective clinical investigations. Ultimately, precision antifungal strategies combined with context-dependent immunomodulation may help shift care from reactive treatment toward earlier, risk-guided intervention, thereby reducing the global burden of virus-associated invasive fungal diseases.
